# Evaluation of IMproving Palliative care Education and Training Using Simulation in Dementia (IMPETUS-D) a staff simulation training intervention to improve palliative care of people with advanced dementia living in nursing homes: a cluster randomised controlled trial

**DOI:** 10.1186/s12877-022-02809-x

**Published:** 2022-02-14

**Authors:** Joanne Tropea, Debra Nestel, Christina Johnson, Barbara J. Hayes, Anastasia F. Hutchinson, Caroline Brand, Brian H. Le, Irene Blackberry, Gideon A. Caplan, Ross Bicknell, Graham Hepworth, Wen K. Lim

**Affiliations:** 1 Department of Medicine and Aged Care, Royal Melbourne Hospital, 6 North Main building, 300 Grattan Street, Parkville, VIC 3050 Australia; 2 Department of Medicine – Royal Melbourne Hospital, University of Melbourne, Parkville, VIC 3010 Australia; 3 School of Clinical Sciences, Monash University, Clayton, VIC 3168 Australia; 4 Austin Precinct, Department of Surgery, University of Melbourne, Parkville, VIC 3010 Australia; 5 Monash Doctors Education, Monash Health, Monash Medical Centre, 246 Clayton Rd, Clayton, VIC 3168 Australia; 6 Faculty of Medicine, Nursing and Health Sciences, Monash University, Monash Medical Centre, 246 Clayton Rd, Clayton, VIC 3168 Australia; 7 Department of Cancer Services, Northern Health Bundoora, 1231 Plenty Road, Bundoora, VIC 3083 Australia; 8 Northern Clinical School, University of Melbourne, Parkville, VIC 3010 Australia; 9 School of Nursing and Midwifery, Deakin University, 221 Burwood Hwy, Burwood, VIC 3125 Australia; 10 Department of Epidemiology and Preventive Medicine, Monash University, 553 St Kilda Road, Melbourne, VIC 3004 Australia; 11 Department of Palliative Care, Royal Melbourne Hospital, 300 Grattan Street, Parkville, VIC 3050 Australia; 12 John Richards Centre for Rural Ageing Research, La Trobe Rural Health School, La Trobe University, Albury-Wodonga Campus, 133 McKoy Street, West Wodonga, VIC 3690 Australia; 13 Prince of Wales Hospital, 320-346 Barker Road, Randwick, NSW 2031 Australia; 14 Prince of Wales Clinical School, UNSW Medicine, University of New South Wales, Sydney, NSW 2052 Australia; 15 Statistical Consulting Centre, University of Melbourne, Parkville, VIC 3010 Australia

**Keywords:** End-of-life care, Palliative care, Dementia, Staff training, Nursing homes

## Abstract

**Background:**

People with dementia have unique palliative and end-of-life needs. However, access to quality palliative and end-of-life care for people with dementia living in nursing homes is often suboptimal. There is a recognised need for nursing home staff training in dementia-specific palliative care to equip them with knowledge and skills to deliver high quality care.

**Objective:**

The primary aim was to evaluate the effectiveness of a simulation training intervention (IMPETUS-D) aimed at nursing home staff on reducing unplanned transfers to hospital and/or deaths in hospital among residents living with dementia.

**Design:**

Cluster randomised controlled trial of nursing homes with process evaluation conducted alongside.

**Subjects & setting:**

One thousand three hundred four people with dementia living in 24 nursing homes (12 intervention/12 control) in three Australian cities, their families and direct care staff.

**Methods:**

Randomisation was conducted at the level of the nursing home (cluster). The allocation sequence was generated by an independent statistician using a computer-generated allocation sequence.

Staff from intervention nursing homes had access to the IMPETUS-D training intervention, and staff from control nursing homes had access to usual training opportunities. The predicted primary outcome measure was a 20% reduction in the proportion of people with dementia who had an unplanned transfer to hospital and/or death in hospital at 6-months follow-up in the intervention nursing homes compared to the control nursing homes.

**Results:**

At 6-months follow-up, 128 (21.1%) people with dementia from the intervention group had an unplanned transfer or death in hospital compared to 132 (19.0%) residents from the control group; odds ratio 1.14 (95% CI, 0.82-1.59). There were suboptimal levels of staff participation in the training intervention and several barriers to participation identified.

**Conclusion:**

This study of a dementia-specific palliative care staff training intervention found no difference in the proportion of residents with dementia who had an unplanned hospital transfer. Implementation of the intervention was challenging and likely did not achieve adequate staff coverage to improve staff practice or resident outcomes.

**Trial registration:**

Australian New Zealand Clinical Trials Registry (ANZCTR): ACTRN12618002012257. Registered 14 December 2018.

**Supplementary Information:**

The online version contains supplementary material available at 10.1186/s12877-022-02809-x.

## Background

People with advanced dementia and their families have specific palliative and end-of-life care needs due to the unpredictable disease trajectory, the severe cognitive impairment that affects the person’s decision-making capacity, verbal communication and behavioural and psychological symptoms of dementia, and the impact it can have on family carer burden [1–5]. For many people with advanced dementia, the last months or years of life are spent in nursing homes [6]. However, there are reports of suboptimal palliative and end-of-life dementia care, including unnecessary transfers to hospital, burdensome interventions, and poor pain management [3, 7, 8]. It has been identified that a major contributory factor to this includes low levels of staff education and skills in provision of palliative and end-of-life care [9, 10].

Training nursing home staff in dementia-specific palliative care is a recognised strategy to improve the quality of care provided, and has been recommended by the European Association for Palliative Care, the Worldwide Hospice Palliative Care Alliance, and more recently in Australia by the Royal Commission into Aged Care Quality and Safety [5, 9–11]. Despite this recommendation, there is a paucity of robust research and evidence to support the use of dementia palliative care training interventions. Previous studies were small, involved one or two sites only, or used a non-randomised study design [12–17]. Our study set out to help fill this gap in evidence and was innovative in its use of simulation training. Simulation is an immersive training technique that attempts to evoke real-life scenarios to promote deeper learning and better transfer of skills to work. There is a growing evidence base to support the benefits of simulation in healthcare education. Meta-analyses have shown simulation using virtual patients and technology enhanced simulation can improve healthcare professionals’ knowledge and skills and have small to moderate patient benefits compared to no training or traditional education methods [18–20]. However, to date very few simulation studies have been conducted in nursing homes and there are no controlled trials on dementia-specific palliative care [21]. Among the few studies that have assessed dementia-specific palliative care training in nursing homes, two used simulation techniques but were single-site, non- randomised studies and of low methodological quality [15, 16].

This study evaluated a training program IMproving Palliative care Education and Training Using Simulation in Dementia (IMPETUS-D) for nurses and personal care workers (PCWs), aimed at improving the quality of palliative care provision and outcomes for people with dementia living in nursing homes. The study used a cluster randomised controlled trial (RCT) design in parallel with a process evaluation. The primary aim was to evaluate the effectiveness of IMPETUS-D training on reducing unplanned transfers to hospital and/or deaths in hospital among residents living with dementia. The study tested the hypothesis that nursing homes that participated in the IMPETUS-D training intervention would reduce the rate of unplanned transfers to hospital and or deaths in hospital among residents with dementia by 20% compared to the control nursing homes.

## Methods

The trial was registered (ANZCTR: ACTRN12618002012257) and conducted according to a pre-defined published protocol to minimise potential biases, and subsequent deviations are reported [22]. Research methods and reporting were in accordance with the CONSORT 2010 statement: extension to cluster randomised trials [23]. Ethics approval was received by the Melbourne Health Human Research and Ethics Committee (HREC/17/MH/336).

### Trial design

A multi-centre, cluster RCT design with an associated process evaluation was undertaken in Australia. Study sites were nursing homes (the clusters). A single private aged care provider managed all facilities. The study ran from December 2018 to October 2020. All nursing homes entered the study in December 2018 and baseline data collection commenced, staff from all the intervention nursing homes received access to the training intervention in early April 2019, the follow-up period commenced 1 October 2019 to 30 September 2020.

### Participants and recruitment

The study involved 24 private nursing homes in Sydney, Melbourne and Adelaide, Australia. To be eligible for inclusion in the study, nursing homes had to have a minimum of 20 people with dementia living permanently in the care home and requiring high level care. All permanent care home residents with a recorded diagnosis of dementia were included.

People with dementia were defined as having a diagnosis of dementia if this was documented in residents’ nursing home files by a medical practitioner or nurse. Nurses and PCWs who worked at the nursing homes were recruited to participate, with additional promotion and information about the study provided face-to-face at staff meetings, and via flyers in staff rooms. Plain language statements were provided in hard copy and electronically to all potential staff participants, and consent was obtained from staff who participated in surveys and interviews. Participation in research activities and the training was voluntary.

Bereaved family members of residents with dementia who died during the follow-up period were invited to participate in a survey. The invitation was posted 6-8 weeks after the death of a resident. If no response was received, one follow-up phone call was made to check the survey had been received, to further explain the project and remind families to participate if they wished.

### Intervention

The IMPETUS-D training program (intervention) was developed for the study by experts in simulation and healthcare worker education. The topics covered were informed by the best practice dementia and palliative care literature, the IMPETUS-D Project Working and Advisory Group, and input from key stakeholders including the end-users (personal care workers and nurses) and family carers. The program comprised 11 modules covering key aspects of best practice palliative and end-of-life care for people with dementia living in nursing homes (Table 1). Modules included case studies with short video scenarios filmed in authentic care home settings. These videos were used as the basis for questions to stimulate learning and practicing ‘what to say’ in different situations, for example, when family are worried about a resident not eating towards the end of life. The length of modules ranged from 15 to 45 mins.

**Table 1:** Modules and topics covered

• Module 1 describes the natural progression of dementia and how to recognise when deterioration is due to dementia versus delirium.	
• Module 2 focuses on caring for a person with advanced dementia when they are approaching the end of their life.	
• Module 3 discusses the reasons why people with advanced dementia are typically best cared for in the care home and describes the challenges people with advanced dementia face in the busy hospital environment.	
• Module 4 introduces Goals of Care (GOC) plans, and highlights that finding out what’s important to the person is vital to providing best care.	
• Module 5 describes what is needed for high quality and best practice GOC plan.	
• Module 6 discusses pain and the challenges in managing pain in people with advanced dementia.	
• Module 7 focuses on recognising the dying phase, including reduced eating and drinking, and helping families understand that reduced eating and drinking at the end of life is normal.	
• Module 8 discusses focusing on what is most important to the person when they are dying and ways to keep a person as comfortable as possible. It also describes terminal restlessness a common end of life symptom, and how care staff can recognise and manage it.	
• Module 9 focuses on changes in breathing at end of life, what to expect as death approaches and ways to support the family. It also discusses the concerns care staff may have as a resident approaches death and includes reflection of learners’ real experiences.	
• Module 10 raises the importance of communicating openly, honestly and frequently with residents and their family members.	
• Module 11 shifts the focus of caring from residents to care providers. It encourages carers to consider their own feelings about death and dying and how they manage these feelings when a resident is at the end of their life and then dies.	

The training could be accessed online via desktop computer, laptop, tablet, or smartphone. PCWs were expected to complete five core modules – Modules 1-3 and 10, and one symptom management module (Modules 6-9). The remaining modules were recommended. Nurses were expected to complete the two goals of care (GOC) modules and the communication module, and the remaining modules were recommended. As an incentive staff were paid the equivalent of three hours of their salary if they participated in the training outside their work hours, and other evidence-based strategies were used to promote staff participation (Table 2).

**Table 2:** Strategies used to encourage staff recruitment and participation in IMPETUS-D training

• Project nurse consultants employed by the provider were recruited in each city to assist with implementation and data collection activities	
• General managers, nurses and PCWs were sent emails by the research team and the provider executive to inform them about the project, and flyers were put up on staff noticeboards at each care home	
• Staff were paid for their time if they participated in the training (face-to-face sessions or online)	
• The research team held face-to-face sessions at each care home	
• Project nurse consultants and / or research team visited nursing homes to promote the training and spoke with staff in-person individually, in small groups, or at staff meetings	
• Other incentive: the nursing home with the highest proportion of staff to participate in the training won a food hamper	

### Control

Staff from nursing homes in the control group did not receive the IMPETUS-D training but had access to usual training opportunities.

### Outcomes

Outcome measures pertain to the cluster or nursing home level. The primary outcome measure was the proportion of residents with dementia who had an unplanned hospital transfer and/or death in hospital during the 6-months follow up. Secondary outcomes measures were: hospital transfers or deaths in hospital over 12-months among residents with dementia, uptake of GOC plans over 6 and 12-months for residents with dementia, change in staff knowledge and attitudes about palliative care for people with dementia at 6 months follow up, and bereaved family satisfaction with care.

### Randomisation and blinding

Randomisation was conducted at the level of the nursing home (cluster). The allocation sequence was generated by an independent statistician using a computer-generated allocation sequence. To ensure equal numbers of nursing homes per study arm, the randomisation used blocks of 4, stratified by city and facility size (small or large, based on the total number of residents). To preserve blinding, the randomisation list was stored and maintained in a secure location by the statistician who was independent from trial recruitment or training.

### Data collection

Details of the data collection for outcome measures can be found in the protocol paper [22]. A dedicated study database was developed using the Research Electronic Data Capture (REDCap) tools hosted at the University of Melbourne [24]. Baseline resident demographic and clinical information, and follow-up resident-level outcomes (hospital transfers and deaths) were collected by project nurse consultants (not blinded to allocation) from the residents’ care home records or extracted from the provider’s database.

At baseline and 6-months follow-up, care home staff were invited to complete a survey which included demographic information and the Questionnaire on Palliative Care for Advanced Dementia (qPAD) [25]. The qPAD is a 2-part instrument consisting of a 23-item knowledge test and 12-item attitude scale. It was used to measure the secondary outcomes of staff knowledge and attitudes towards providing palliative dementia care. A total qPAD score (range 12-83) was derived by adding knowledge test (range 0-23) and attitude scale (range 12-60) scores; higher scores indicating better knowledge and more positive attitudes.

During the follow-up period, bereaved families’ perception of care was assessed using the Satisfaction With Care at the End-of-Life Dementia (SWC-EOLD) scale [26]. The SWC-EOLD assessed satisfaction with care during the last 90 days of life, and consisted of 10 items, each measured on a 4-point Likert scale defined as 1=strongly disagree, to 4=strongly agree. The total SWC-EOLD score was calculated by adding all 10 items (range 10-40); higher scores indicated greater satisfaction.

### Sample size and power calculations

An a-priori sample size calculation was undertaken as per protocol [22]. The power analyses were based on the following assumptions: proportion of composite events (hospital transfer and/or death in hospital) of 0.65 and 0.85 over 6 months of follow-up in the intervention and control groups respectively, two-sided significance level of 0.05 (alpha). We estimated that 12 clusters (nursing homes) in each study arm, with 20 – 25 residents with dementia in each cluster, was needed with an Intra-Cluster Correlation (ICC) of 0.05. With these assumptions, the minimum power to observe a difference of proportion by 0.20 between the intervention and control group is 94%.

### Statistical analysis

Descriptive statistics were used to summarise baseline care home and resident-level characteristics. Analyses of primary and secondary outcomes were on an intention to treat (ITT) basis, with the fundamental principles of analysing data from cluster RCTs followed. To compare the proportion of composite events for the primary outcome, logistic regression models were used with standard errors weighted by the cluster effects, clusters being the nursing homes. Estimated odds ratio (OR) and associated 95% confidence interval are presented, along with the p value for testing the hypothesis that the OR is 1. Total scores from the qPAD and SWC-EOLD were considered as a continuous variable. Mean scores were compared between the groups using mixed models which adjust for cluster effect. Statistical analyses were conducted in Stata MP version 15 [27]. All statistical tests were two-sided and the significance level was set at 0.05.

### Process evaluation

Process evaluation was used to assess the implementation of the training intervention, to explore barriers and facilitators that influence implementation of the intervention, as well as the intervention mechanisms and outcomes, and was informed by the Medical Research Council (MRC) Process Evaluation of Complex Interventions [28] to guide the process evaluation and Consolidated Framework for Implementation Research (CFIR) [29, 30]. The CFIR provides a menu of constructs across five domains that have been associated with effective implementation. The CFIR domains of Inner setting, Intervention characteristics, and Characteristics of individuals, were used to explore participants’ experience, and barriers to participation, in addition to measures of staff participation and module completion (reach and dose) as described below.

The a-priori aim was for 80% of staff to complete the core modules for their discipline. This was amended during the training period to 50% of nurses in response to feedback from the provider and from staff that staff had other mandated training occurring at the same time. Data on the number of staff who participated in the training, the number of modules, and the number of core modules commenced and completed was collected from the learning management system (LMS) and from attendance records at each face-to-face training session.

Data on participants’ experience and satisfaction with the intervention was collected from feedback items at the end of each module, and at 6-month follow-up staff interviews and follow-up staff surveys. Data from the research team notes and log of activities, informal feedback from staff, follow-up staff surveys, and staff interviews was used to identify factors that may have influenced reach, dose, participant satisfaction and barriers to participation.

Qualitative data from the surveys and interviews were imported from Microsoft Excel and Microsoft Word files into NVivo 12 for coding [31]. Data was analysed by the research team and key themes identified based on anticipated and emergent themes [32]. An iterative process was used, with coding trees and summarised data presented to members of the IMPETUS-D Project Working and Advisory Group for review and input.

## Results

**Fig. 1:**
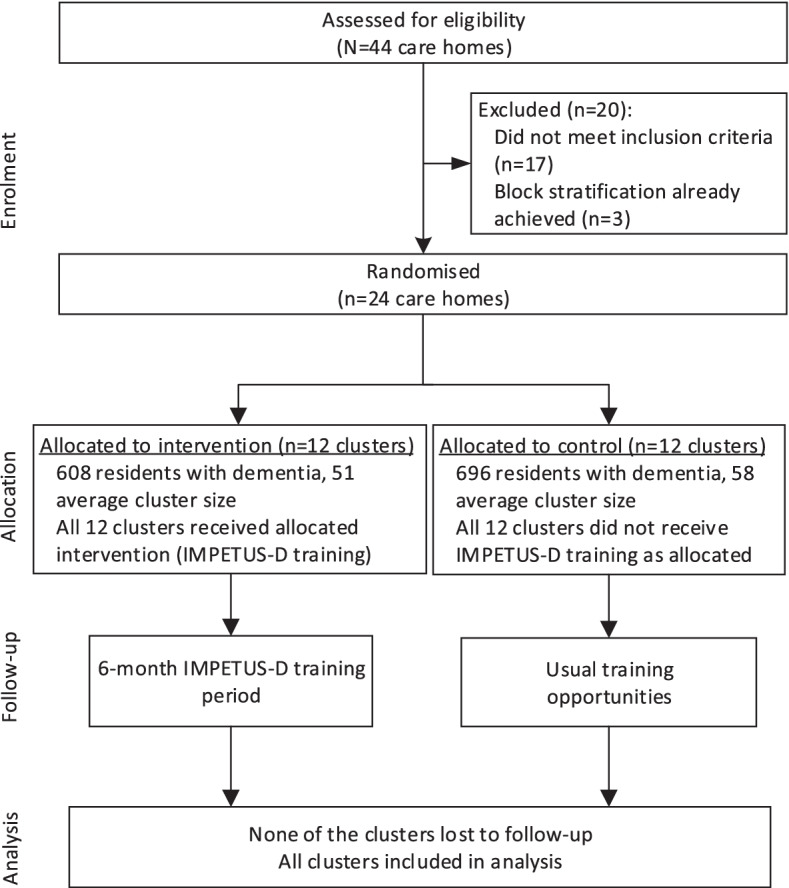
Study flowchart

Of the 44 nursing homes, 27 were eligible for inclusion. To obtain balance across locations and size, 24 nursing homes were randomised into the intervention or control group (Fig. 1). All 24 agreed to participate in the study, and all remained involved in the study throughout the project timeline.

### Residents with dementia

At baseline, among all residents living in the 24 nursing homes 1304 (59.8%) had a diagnosis of dementia recorded; 608 in the intervention nursing homes and 696 in the control nursing homes. Demographic and clinical data of residents with dementia were similar for those living in the intervention and control homes, except more residents from the intervention group had an advance care directive (ACD) in place (Table 3).

**Table 3:** Baseline clinical and demographic characteristics of residents with dementia

Characteristic	Intervention nursing homes (*n* = 608)	Control nursing homes (*n* = 696)	Total (*N* = 1,304)
Age in years, median (IQR)	86.4 (81.0-91.5)	87.3 (80.9-91.5)	86.9 (80.9-91.5)
Female, n (%)	409 (67.3)	463 (66.5)	872 (66.9)
Years living in care home, median (IQR)	2.2 (1.0-4.6)	2.3 (0.9-4.2)	2.3 (0.9-4.4)
ACFI scores - high, n (%)			
Activities of daily living Behaviour Complex health care needs	342 (56.3) 468 (77.0) 416 (68.4)	425 (61.1) 556 (79.9) 502 (72.1)	767 (58.8) 1024 (78.5) 918 (70.4)
ACD in place, n (%)	198 (32.6)	190 (27.3)	388 (29.8)
Type of dementia			
Alzheimer’s disease Vascular dementia Mixed dementia Lewy body dementia Fronto-temporal dementia Other Not specified	284 (46.4) 67 (11.0) 56 (9.2) 14 (2.3) 11 (1.8) 20 (3.3) 158 (26.0)	346 (49.7) 87 (12.5) 53 (7.6) 12 (1.7) 10 (1.4) 30 (4.3) 158 (22.7)	628 (48.2) 154 (11.8) 109 (8.4) 26 (2.0) 21 (1.6) 50 (3.8) 316 (24.2)
Comorbidities			
CHF Chronic lung disease Chronic kidney failure Chronic liver disease Stroke Cancer	68 (11.2) 76 (12.5) 51 (8.4) 6 (1.0) 102 (16.8) 109 (17.9)	92 (13.2) 92 (13.2) 69 (9.9) 4 (0.6) 122 (17.5) 115 (16.5)	160 (12.3) 168 (12.9) 120 (9.2) 10 (0.8) 224 (17.2) 224 (17.2)

*Abbreviations*: *ACD* advance care directive, *ACFI* Aged care funding instrument, *CHF* congestive heart failure, *IQR* inter quartile range

Unplanned hospital transfers and deaths in hospital are shown in Table 4. During 6-months follow-up, a total of 260 (19.9%) residents with dementia from all care homes had at least one unplanned hospital transfer; 128 (21.1%) in the intervention and 132 (19.0%) in the control group. Analysis of the primary outcome (proportion of hospital transfers or deaths in hospital among residents with dementia over 6 months) yielded an odds ratio of 1.14 (95% CI, 0.82-1.59), *p* = 0.44. Overall, 154 residents died during 6-months, 75 (12.3%) from the intervention and 79 (11.4%) from the control group.

**Table 4:** Number and proportion of residents with dementia with an unplanned hospital transfer and death in hospital at 6- and 12-months follow-up (*N* = 1304)

Outcomes, n (%)	Intervention (*n* = 608)	Control (*n* = 696)	Odds ratio (95% CI), *p* value
Hospital transfers, 6 months	128 (21.1)	132 (19.0)	1.14 (0.82 – 1.59), *p* = 0.44
Hospital transfers, 12 months	201 (33.1)	206 (29.6)	1.17 (0.84 – 1.63), *p* = 0.34
Deaths in hospital, 6 months	14 (18.7)	14 (17.7)	1.07 (0.39 – 2.91), *p* = 0.90
Deaths in hospital, 12 months	22 (3.6)	28 (4.0)	0.90 (0.44 – 1.83), *p* = 0.76

During 12-months follow-up, 407 (31.2%) residents with dementia had at least one unplanned transfer to hospital, 201 (33.1%) from the intervention and 206 (29.6%) from the control group (odds ratio 1.17; 95% CI, 0.84-1.63); and 310 (23.8%) residents died, 154 (25.3%) from the intervention and 156 (22.4%) from the control group.

Two of the core nursing modules focused on GOC discussions and documenting GOC medical treatment plans [33]. A new GOC form was introduced in the module and all senior nurses and general managers were sent a copy of the form. However, there was no uptake of the form which relied on a doctor for completion. This was despite general practitioners being sent information about the forms.

### Staff knowledge and attitudes

A total of 330 staff completed the follow-up qPAD, 122 from the intervention and 208 from the control nursing homes. Staff from both groups were similar in terms of sex, age, position, years’ of aged care experience, hours worked per week, and highest level of education. (Appendix 1). Among them, 218 staff completed both baseline and follow-up qPAD, 96 from the intervention and 122 from control nursing homes.

There was no significant between-group difference in total qPAD scores; intervention group mean score=60.1 (95% CI 57.4-62.8) compared to the control group mean score=59.5 (95% CI 57.2-61.8), *p* = 0.77. Most of the variability was contributed by individuals (93%) not nursing homes (7%) indicating clustering did not have a big effect on scores. Similarly, when baseline scores were accounted for, there was no significant between-group difference in total qPAD scores; adjusted intervention group mean score=61.1 and control group mean score=60.0 (*p* = 0.53).

### Bereaved carer survey

A total of 72 of 258 bereaved carer surveys were received over 12-months. At the request of the provider, surveys were not sent if nursing homes had experienced COVID outbreaks or if coroner’s investigation was underway. There were no significant between group differences in mean SWC-EOLD scores, with mean scores of 26.6 in the intervention and 27.0 in the control group, *p* = 0.80. Most of the variability was contributed by individuals (87%) not nursing homes (13%) demonstrating that clustering did not have had a big effect on SWC-EOLD score.

### Process evaluation findings

#### Fidelity

The IMPETUS-D program was largely implemented as intended, with all planned implementation strategies completed in full or in part. Due to low uptake of the online modules and feedback from staff, the training period was extended from 2 months as originally planned to 6 months, and face-to-face training sessions were held by members of the research team and the project consultant nurses at each of the intervention sites to boost training participation. The IMPETUS-D Project Working and Advisory Group decided the priority was to maximise staff participation. Although the mode of delivery of the IMPETUS-D intervention was modified, the module content was not altered and the online modules were projected on a screen or on multiple laptops for group learning.

#### Reach and dose

**Fig. 2:**
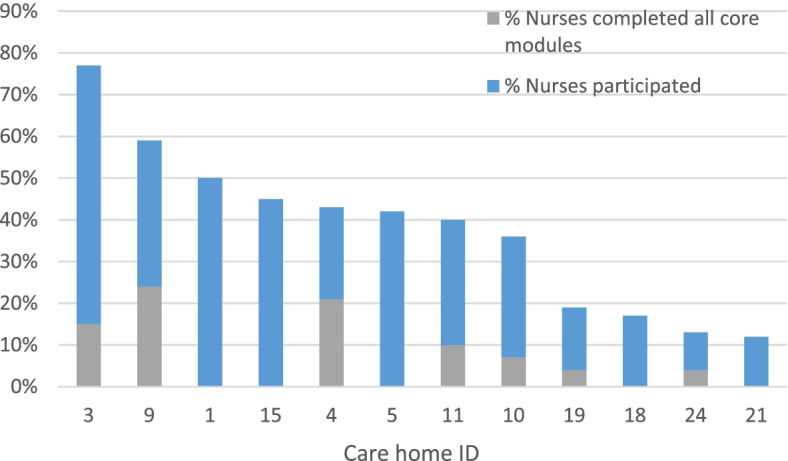
Percentage of nurses who completed core modules or participated by care home

During the 6-month training period 42 (4.3%) staff completed their core modules, 27 (3.7%) PCWs and 15 (6.0%) nurses. None of the nursing homes achieved the aim of 50% of nurses completing core modules. The three nursing homes with the highest proportion of nurses completing core modules were 24% (care home 9), 21% (care home 4) and 15% (care home 3) (Fig. 2).

Overall, 251 (26%) staff completed at least one module during the training period, 79 nurses and 172 PCWs. One hundred sixty staff attended the face-to-face sessions, and 102 participated in the training online, with some staff completing modules both online and at face-to-face sessions. The proportion of staff from each care home who participated in the training ranged from 15 to 53%.

#### Staff satisfaction with the training

Among the 103 follow-up survey respondents who had done the training, 61 (59%) agreed that completing the modules increased their confidence in looking after residents with dementia, 12 (12%) did not agree or were unsure and 30 (29%) did not respond. The reasons staff gave for not participating in the training included: 25% were unable to attend the session, 17% were not aware of the training, 16% had technical issues accessing the training online, and 15% did not have time.

#### Barriers to participation

The CFIR was used to guide the thematic analysis of the qualitative interview and survey data [30]. Barriers were categorized according to the following three CFIR domains and their associated constructs – (i) Inner setting (the organisation of the care home), (ii) Intervention characteristics, and (iii) Characteristics of individuals. Examples of quotes from the interviews and survey data on main barriers (according to CFIR constructs) to staff participation have been presented in Table 5.

**Table 5:** Examples of quotes for key barriers by CFIR domain and construct

CFIR domain and construct	Example of quotes from qualitative data
Inner setting – Available resources	Unless you are covered on the floor and you are able to do your training…during protected time. (Interview with PCW158) Different time sessions as I was on shift every time the sessions were held. (Survey, ID_224)
Inner setting – Leadership engagement	…that perhaps no one’s taking responsibility at the home level to make sure that we’ve got this compliance…(Interview with Project Consultant Supervisor)
Inner setting – Relative priority	… it's not about the topic. It is about the load that we had then… so finding the time and finding um a little bit because the new standards (Interview with GM04)
Intervention characteristics – Complexity and design	Not easily accessible, tried a few times but was issues with log in, incorrect password etc (Survey, ID_499)
Characteristics of individuals (Staff) – Educational preferences	Much prefer to come to a group session, away from home (Group interview, PCW022)

One of the main barriers to staff participation was having competing priorities that occurred at the same time as the IMPETUS-D program was implemented, specifically the introduction of the National Aged Care Standards and the Royal Commission into Aged Care Quality and Safety. Staff were expected to attend training in preparation for the new standards, and many had additional administrative work in relation to the new standards and the Royal Commission, and these activities took time and the focus away from IMPETUS-D. These barriers relate to the CFIR constructs of relative priority (standards took priority over IMPETUS-D training) and available resources (staff not having adequate or dedicated time and resources to do the training). In addition, there were organisational and nursing home level barriers, that affected implementation including variable leadership engagement, intra-organisational communication, turnover of care home management and senior nurse roles at 50% of intervention homes (stability of the team), and time constraints and disruption to schedule. Some characteristics of the training intervention itself were also reported as potential barriers to implementation including the online and individual learning approach, the use of a separate LMS rather than the organisation’s LMS that staff were familiar with, complexity of the intervention with multiple modules, targeting multiple disciplines, and that the training was not mandatory.

## Discussion

The IMPETUS-D simulation-based staff training program on quality palliative and end-of-life care in dementia had no effect on hospital transfers and deaths in hospital of nursing home residents with dementia, staff knowledge and attitudes, or bereaved family satisfaction with care. Implementation of the intervention was suboptimal with only 26% of staff participating in the program.

### Comparison with other studies

Our systematic review of five controlled studies found there is a lack of evidence to support simulation training in non-cancer palliative care for healthcare workers [21]. Improvements in staff communication skills, knowledge acquisition and attitudes towards death and comfort were reported in three studies, but these studies had several methodological limitations. Further, one good quality RCT was unable to show any change in clinician practice or patient outcomes. The variability of these findings highlighted the need for more rigorous research to evaluate the effect of simulation training in this clinical field, including studies to establish optimal duration and types of simulation applications, and the impact of training interventions on practice change and patient outcomes.

Two recent cluster RCTs have evaluated palliative care training programs in nursing homes [34, 35]. However, these studies did not focus on dementia-specific palliative care. The European multi-country PACE study assessed an intervention that used a train-the-trainer approach to integrate non-specialist palliative care in 37 nursing homes compared to 36 controls [35]. They were unable to show improvements in residents’ comfort at end-of-life, and despite improvements in intervention staff knowledge compared to the controls, the difference was not clinically relevant. The process evaluation found high variability in the implementation of the intervention, and reported the main reasons related to the program itself and its delivery, the PACE trainer, and organisational issues [36]. This study, like the IMPETUS-D study did not have effective implementation of the intervention.

The study by Lamppu et al. (2021) assessed the effectiveness of staff training in palliative and end-of-life care in 20 Helsinki nursing homes [34]. The intervention training was based on a palliative care training-needs survey and this likely contributed to the high participation rates (74%). However, they were unable to show any between-group differences in residents’ health-related quality-of-life, hospital admissions or emergency presentations. They attributed the null findings to potential poor selection of quality-of-life instrument, lower than expected deaths, and different learning needs of professions; and concluded that training interventions alone might be insufficient to produce improvements in practice or outcomes.

Both these studies were unable to show the training led to improvements in resident outcomes. Further studies may need to incorporate interventions beyond education alone to impact on staff practice change and improve resident and family carer outcomes. There is some evidence to suggest use of staff training in combination with follow-up expert consultation [37] or supervision sessions to help staff incorporate strategies into practice [38], but this has not been well established for staff training in aged care or dementia-specific palliative care.

### Strengths and limitations

Strengths of the IMPETUS-D study include its large sample size. multi-site design and use of an innovative simulation-enhanced program about the specific palliative care needs of people with dementia. All 24 nursing homes recruited to the study, participated in the research and all intervention nursing homes participated in the training. However, there were several study limitations.

Despite use of evidence-based implementation strategies, the degree of staff participation was lower than expected and a key limitation to this study. One of the biggest issues was the timing of the implementation of the intervention. It was unfortunate that the training was implemented at the same time as major changes in the national aged care standards were introduced and these took priority. Despite nursing homes being aware of the new standards, they were not prepared for the amount of additional training and administrative work it entailed. Several other barriers to IMPETUS-D uptake were identified and related to staff (turnover and high workload), organisational or inner setting factors (lack of time, communication, scheduling disruptions), and the characteristics of the training intervention (technical issues, its voluntary nature, complexity and design quality). Strategies such as having the IMPETUS-D training integrated into the organisation’s training schedule and available on the organisation’s learning management system, or implementing a more simplified version such as targeting only nurses or implementing it in a stepped approach may have helped overcome some of these barriers and increased uptake. However, many of these challenges have been reported in other nursing home-based studies [39, 40], and highlight the need for future research to evaluate the effect of strategies to address common barriers.

A pilot intervention phase was not undertaken due to time and resource constraints. This may have aided identification of modifiable barriers to participation and strategies to overcome these barriers could have been put in place. However, a pilot phase may not have mitigated unexpected system changes which were beyond researcher control. Further, the investigators considered that the bespoke design of the training package and high-level stakeholder buy-in and engagement with the training package with all homes being managed by the same private aged care provider would support staff access to and uptake of training. Unfortunately, in this study, this was not sufficient in and of itself to ensure staff participation and further research is needed to better understand nursing home context specific barriers and strategies to overcome these.

This study set out to evaluate the effectiveness of the IMPETUS-D intervention, but without sufficient staff participation (implementation failure), we were unable to establish the true effect of the training on staff knowledge, practice, and resident outcomes. Future work in this area might benefit from a more detailed assessment of individual care homes’ readiness to implement the training with input from general managers, nurses, PCWs, residents, and their families. This information could then be used to target care homes that are ready to implement the training, and to inform the research or implementation team on how best to support implementation, based on the local needs.

## Conclusion

Our study of a staff training program on palliative dementia care was unable to show any differences in hospital transfers or staff knowledge and attitudes. Staff participation was lower than expected and highlighted the need to ensure nursing homes are ready to implement the intervention. Training programs for nursing home staff are important and necessary as the population ages and the prevalence of dementia increases. Further research is warranted to assess the impact of a more simplified and sustainable version of the IMPETUS-D training intervention, for example by assessing a smaller number of modules and targeting fewer staff groups; and to establish facilitation strategies that work to increase staff participation and successfully influence implementation.

## Supplementary Information

**Additional file 1: Appendix 1.** Characteristics and qPAD scores of staff who completed the follow-up survey (*N*=330)

## Data Availability

Data will be available after analyses is finalised and report / publication has been submitted and approved. Unidentifiable individual participant data and related data dictionaries will be available. Access is subject to approval by the Principal Investigator Professor Wen Kwang Lim.

## Abbreviations

ACD: Advance care directive
ACFI: Aged Care Funding Instrument
ANZCTR: Australia and New Zealand Clinical Trial Register
CFIR: Consolidated Framework for Implementation Research
GOC: Goals of Care
HREC: Human Research and Ethics Committee
ICC: Intra-Cluster Correlation
IMPETUS-D: IMproving Palliative care Education and Training Using Simulation in Dementia
ITT: Intention to treat
LMS: Learning management system
OR: Odds ratio
PCWs: Personal care workers
RCT: Randomised controlled trial
