# Supplementary material for: Evaluation of IMproving Palliative care Education and Training Using Simulation in Dementia (IMPETUS-D) a staff simulation training intervention to improve palliative care of people with advanced dementia living in nursing homes: a cluster randomised controlled trial
Source: BMC Geriatr. 2022 Feb 14;22:127. doi: 10.1186/s12877-022-02809-x (PMC8845393; doi:10.1186/s12877-022-02809-x)
Supplement: Supplementary file 1 — Additional file 1: Appendix 1. Characteristics and qPAD scores of staff who completed the follow-up survey (N=330) [file 12877_2022_2809_MOESM1_ESM.docx]

# SUPPLEMENTARY MATERIAL

Evaluation of Improving Palliative care Education and Training Using Simulation in Dementia (IMPETUS-D) in nursing homes: a cluster randomised controlled trial

**Appendix 1: Characteristics and qPAD scores of staff who completed the follow-up survey (N=330)**

| **Characteristics, n (%)** | **Staff from intervention nursing homes (n=122)** | **Staff from control nursing homes (n=208)** |
| --- | --- | --- |
| City  Sydney  Adelaide  Melbourne | 31 (25)  33 (27)  58 (48) | 42 (20)  104 (50)  62 (30) |
| Position  Registered nurse  Enrolled nurse  Care worker  Other | 17 (13)  6 (5)  97 (80)  2 (2) | 36 (17)  6 (3)  158 (76)  8 (4) |
| Age in years, median (IQR) | 40 (32, 52) | 42 (32, 52) |
| Sex  Female  Male  Other/missing | 100 (82)  6 (5)  16 (13) | 119 (57)  16 (8)  73 (35) |
| Highest level of education  Lower or upper secondary school  Post-secondary, non-tertiary course  University – bachelor degree  University – postgraduate degree  Other  Missing | 33 (27)  28 (23)  32 (26)  12 (10)  4 (3)  13 (11) | 58 (28)  45 (22)  63 (30)  21 (10)  4 (2)  17 (8) |
| Hours worked per week, median (IQR) | 30 (20, 35) | 32.5 (22, 37.5) |
| Years worked at current care home  Less than 5 years  5-10 years  11-15 years  More than 15 years  Missing | 67 (55)  21 (17)  11 (9)  12 (10)  11 (9) | 98 (47)  64 (31)  22 (11)  11 (5)  13 (6) |
| Years worked in residential care  Less than 5 years  5-10 years  11-15 years  More than 15 years  Missing | 57 (47)  23 (19)  14 (11)  20 (16)  8 (7) | 81 (39)  67 (32)  26 (13)  23 (11)  11 (5) |
| Questionnaire on Palliative care for Advanced Dementia, median (IQR)  Knowledge test  Attitude scale  Total score | 15 (13, 17)  45 (42, 49)  61 (56, 66) | 16 (14, 18)  46.5 (41, 50.5)  61 (55.5, 67) |

Abbreviations: IQR interquartile range; qPAD Questionnaire on Palliative Care for Advanced Dementia
